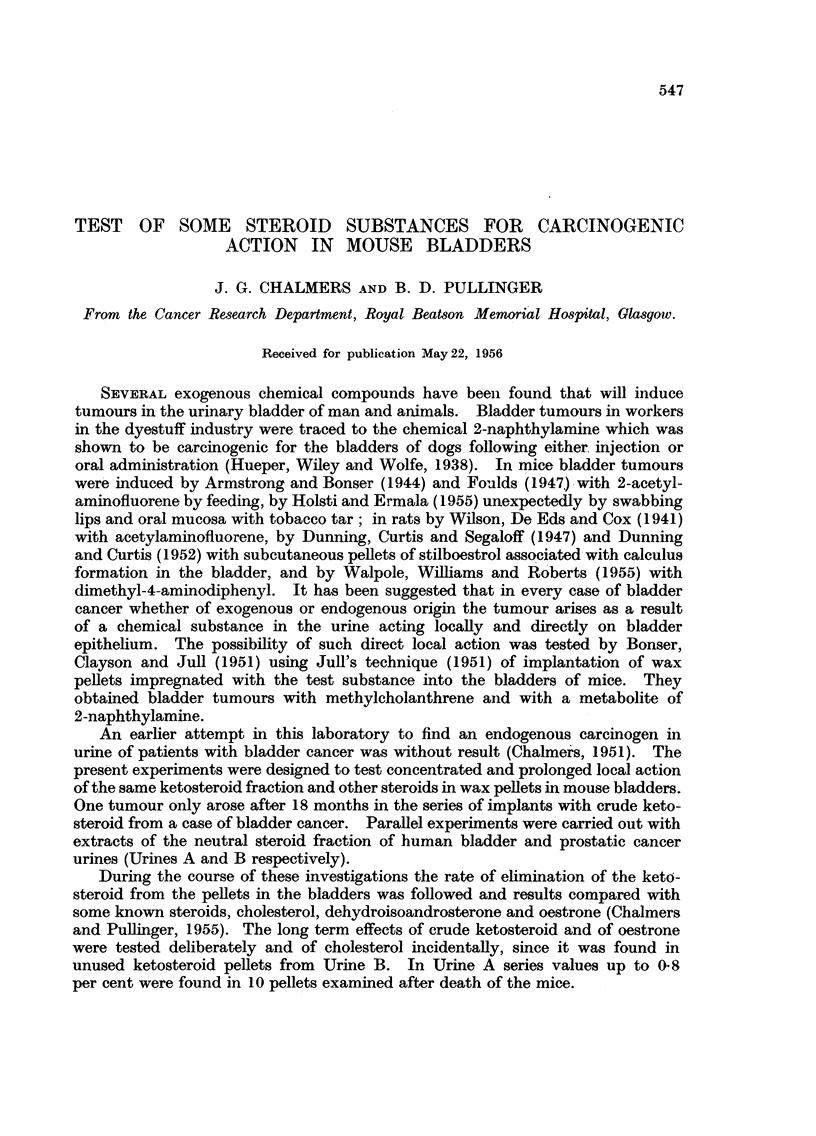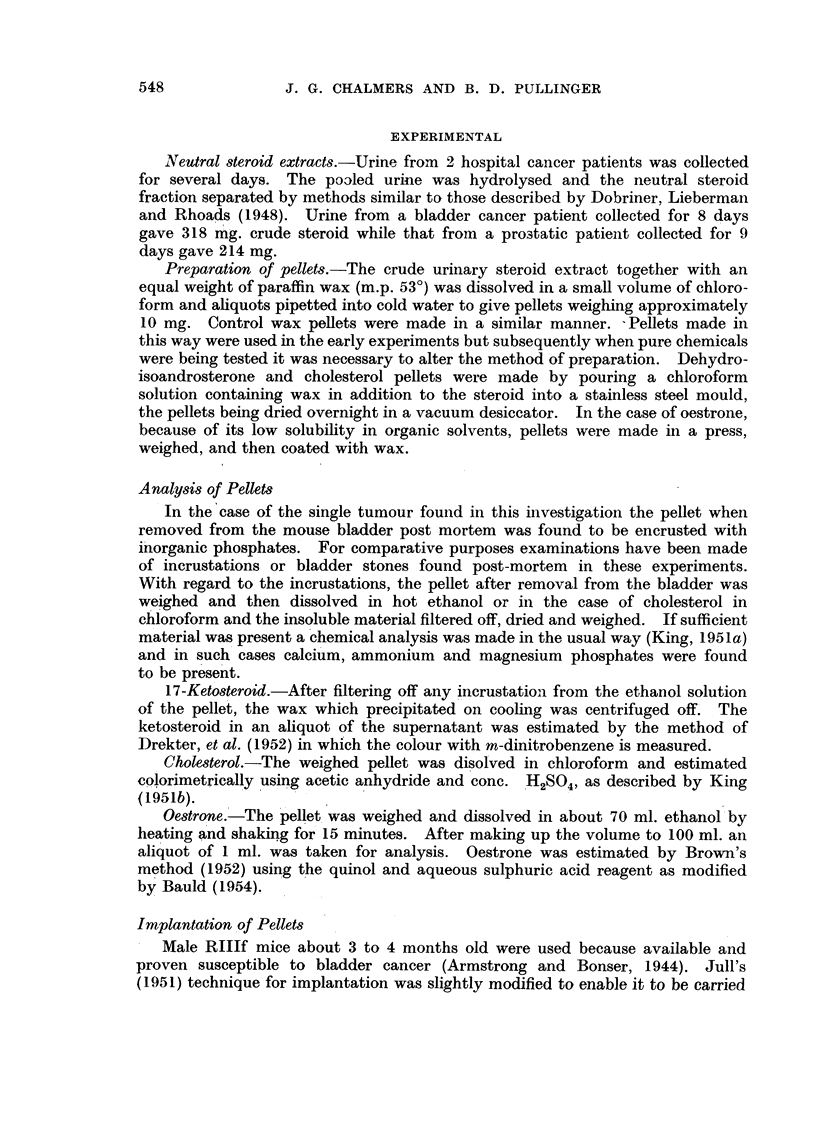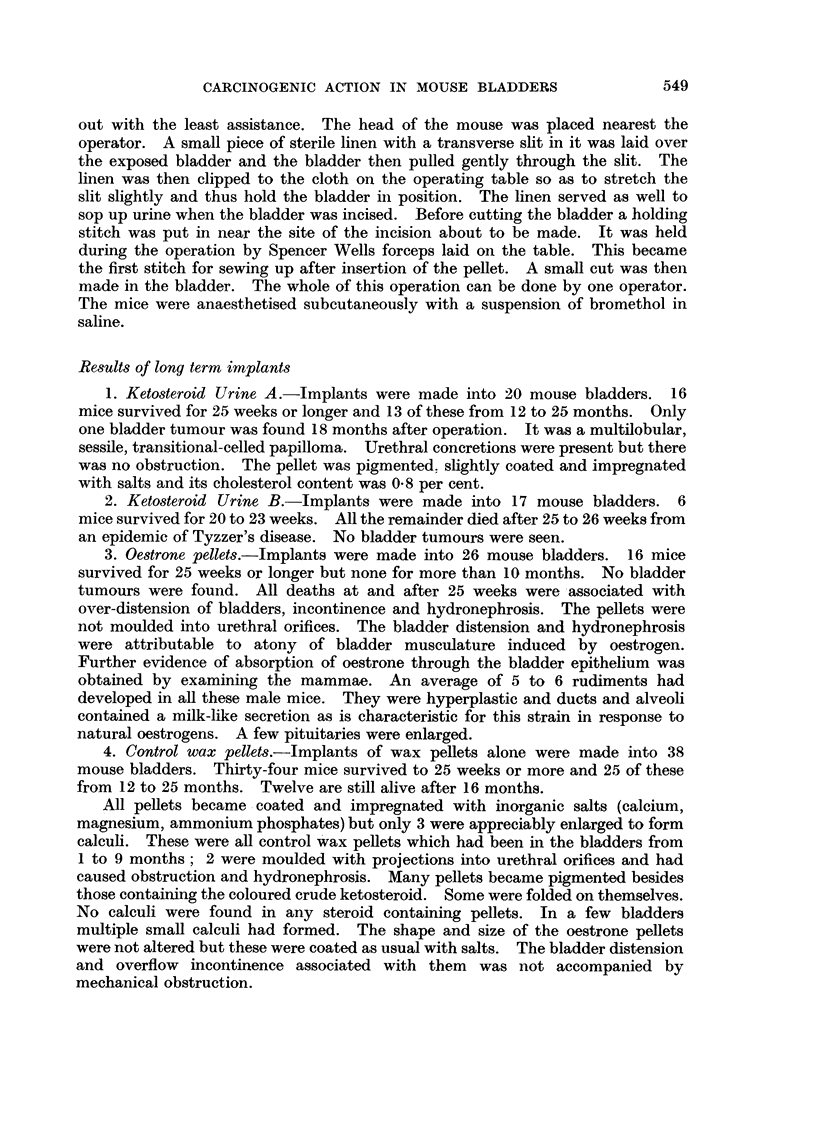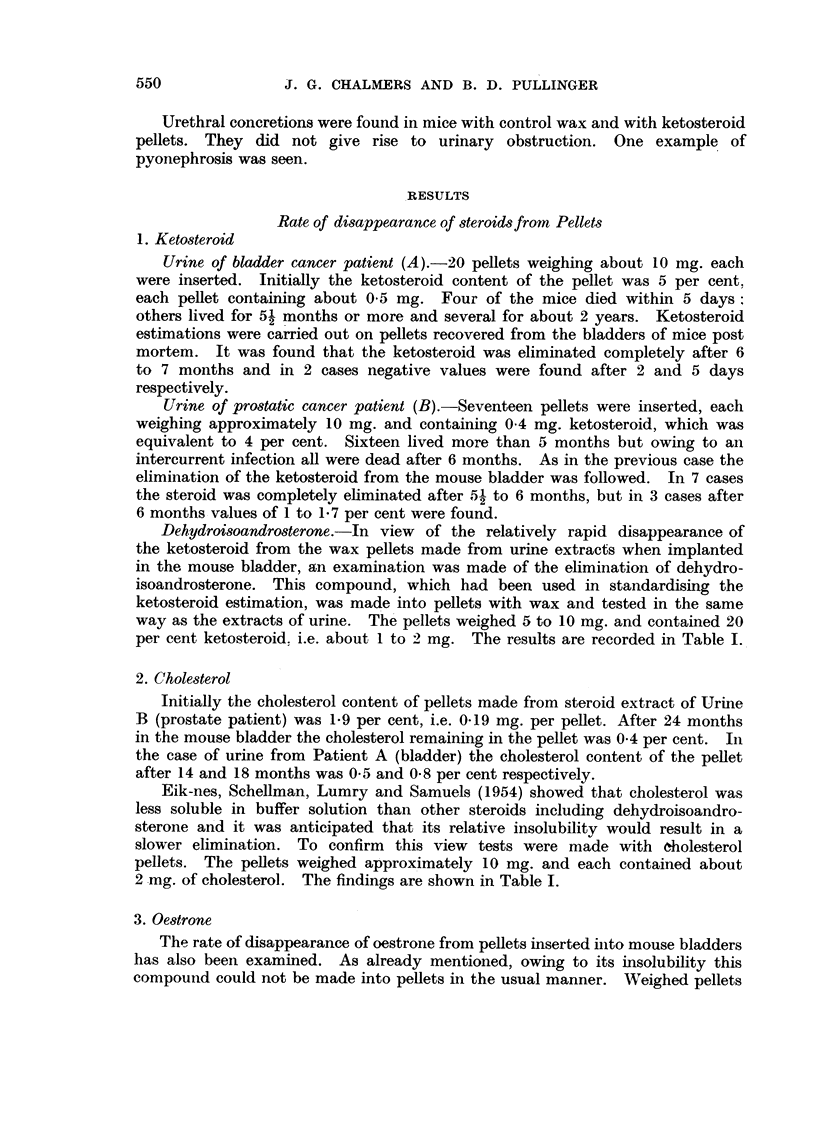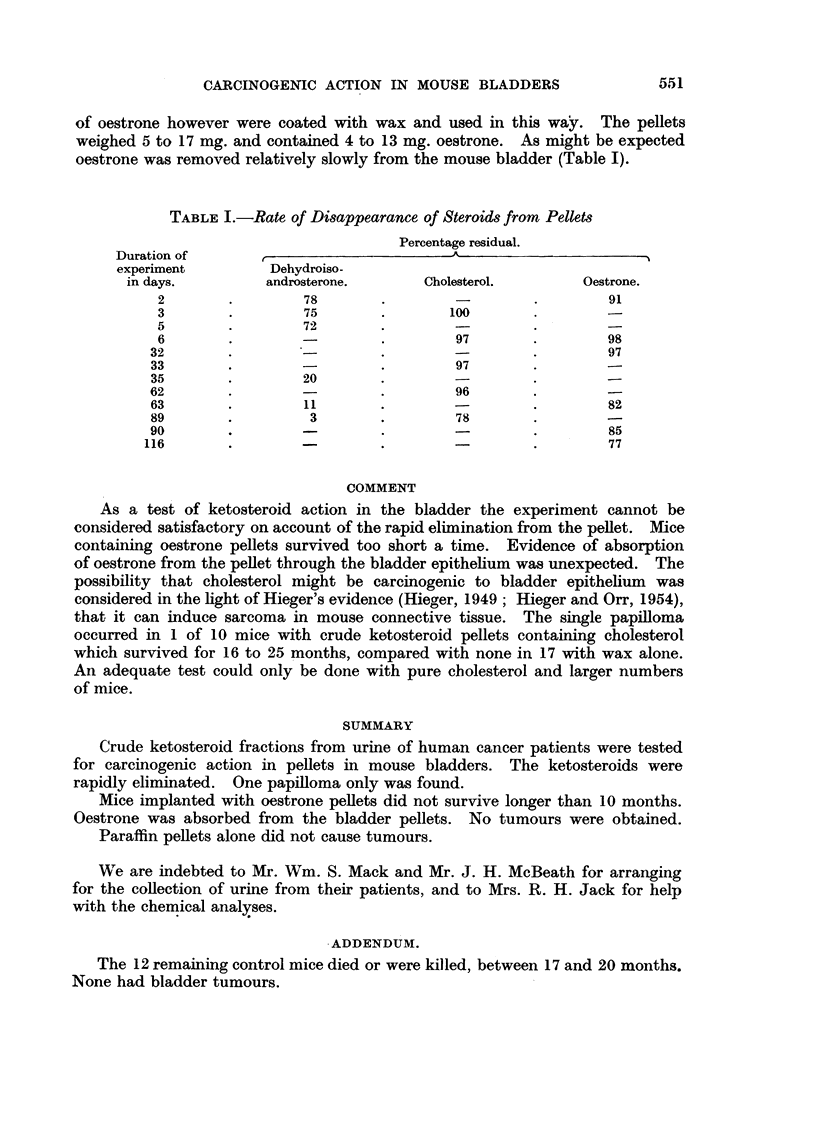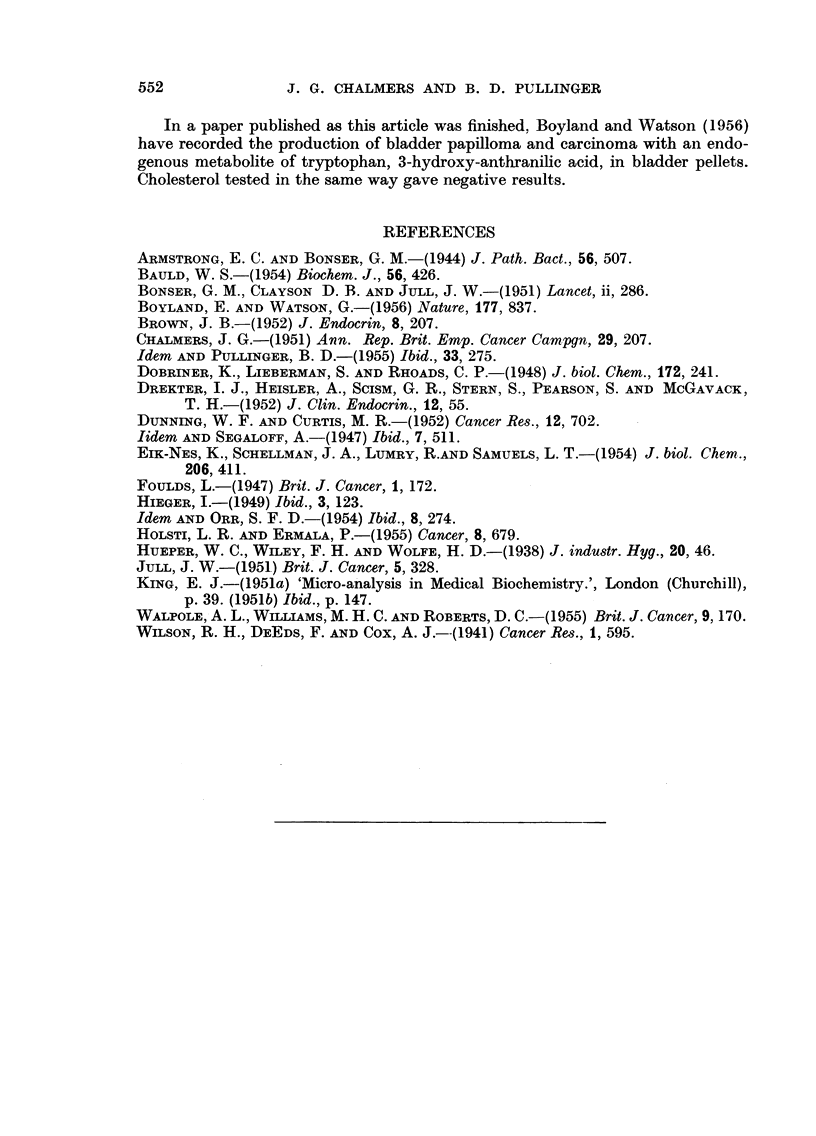# Test of Some Steroid Substances for Carcinogenic Action in Mouse Bladders

**DOI:** 10.1038/bjc.1956.64

**Published:** 1956-09

**Authors:** J. G. Chalmers, B. D. Pullinger


### TEST OF SOME STEROID SUBSTANCES FOR CARCINOGENIC ACTION IN MOUSE BLADDERS

J. G. CHALMERS AND B. D. PULLINGER

*From the Cancer Research Department, Royal Beatson Memorial Hospital, Glasgow.*

Received for publication May 22, 1956

SEVERAL exogenous chemical compounds have been found that will induce tumours in the urinary bladder of man and animals. Bladder tumours in workers in the dyestuff industry were traced to the chemical 2-naphthylamine which was shown to be carcinogenic for the bladders of dogs following either injection or oral administration (Hueper, Wiley and Wolfe, 1938). In mice bladder tumours were induced by Armstrong and Bonser (1944) and Foulds (1947) with 2-acetyl-aminofluorene by feeding, by Holsti and Ermala (1955) unexpectedly by swabbing lips and oral mucosa with tobacco tar ; in rats by Wilson, De Eds and Cox (1941) with acetylaminofluorene, by Dunning, Curtis and Segaloff (1947) and Dunning and Curtis (1952) with subcutaneous pellets of stilboestrol associated with calculus formation in the bladder, and by Walpole, Williams and Roberts (1955) with dimethyl-4-aminodiphenyl. It has been suggested that in every case of bladder cancer whether of exogenous or endogenous origin the tumour arises as a result of a chemical substance in the urine acting locally and directly on bladder epithelium. The possibility of such direct local action was tested by Bonser, Clayson and Jull (1951) using Jull's technique (1951) of implantation of wax pellets impregnated with the test substance into the bladders of mice. They obtained bladder tumours with methylcholanthrene and with a metabolite of 2-naphthylamine.

An earlier attempt in this laboratory to find an endogenous carcinogen in urine of patients with bladder cancer was without result (Chalmers, 1951). The present experiments were designed to test concentrated and prolonged local action of the same ketosteroid fraction and other steroids in wax pellets in mouse bladders. One tumour only arose after 18 months in the series of implants with crude ketosteroid from a case of bladder cancer. Parallel experiments were carried out with extracts of the neutral steroid fraction of human bladder and prostatic cancer urines (Urines A and B respectively).

During the course of these investigations the rate of elimination of the ketosteroid from the pellets in the bladders was followed and results compared with some known steroids, cholesterol, dehydroisoandrosterone and oestrone (Chalmers and Pullinger, 1955). The long term effects of crude ketosteroid and of oestrone were tested deliberately and of cholesterol incidentally, since it was found in unused ketosteroid pellets from Urine B. In Urine A series values up to 0·8 per cent were found in 10 pellets examined after death of the mice.

### EXPERIMENTAL

*Neutral steroid extracts.*—Urine from 2 hospital cancer patients was collected for several days. The pooled urine was hydrolysed and the neutral steroid fraction separated by methods similar to those described by Dobriner, Lieberman and Rhoads (1948). Urine from a bladder cancer patient collected for 8 days gave 318 mg. crude steroid while that from a prostatic patient collected for 9 days gave 214 mg.

*Preparation of pellets.*—The crude urinary steroid extract together with an equal weight of paraffin wax (m.p. 53°) was dissolved in a small volume of chloroform and aliquots pipetted into cold water to give pellets weighing approximately 10 mg. Control wax pellets were made in a similar manner. Pellets made in this way were used in the early experiments but subsequently when pure chemicals were being tested it was necessary to alter the method of preparation. Dehydroisoandrosterone and cholesterol pellets were made by pouring a chloroform solution containing wax in addition to the steroid into a stainless steel mould, the pellets being dried overnight in a vacuum desiccator. In the case of oestrone, because of its low solubility in organic solvents, pellets were made in a press, weighed, and then coated with wax.

### *Analysis of Pellets*

In the case of the single tumour found in this investigation the pellet when removed from the mouse bladder post mortem was found to be encrusted with inorganic phosphates. For comparative purposes examinations have been made of incrustations or bladder stones found post-mortem in these experiments. With regard to the incrustations, the pellet after removal from the bladder was weighed and then dissolved in hot ethanol or in the case of cholesterol in chloroform and the insoluble material filtered off, dried and weighed. If sufficient material was present a chemical analysis was made in the usual way (King, 1951*a*) and in such cases calcium, ammonium and magnesium phosphates were found to be present.

17-*Ketosteroid.*—After filtering off any incrustation from the ethanol solution of the pellet, the wax which precipitated on cooling was centrifuged off. The ketosteroid in an aliquot of the supernatant was estimated by the method of Drekter, *et al.* (1952) in which the colour with *m*-dinitrobenzene is measured.

*Cholesterol.*—The weighed pellet was disolved in chloroform and estimated colorimetrically using acetic anhydride and conc. $H_2SO_4$, as described by King (1951*b*).

*Oestrone.*—The pellet was weighed and dissolved in about 70 ml. ethanol by heating and shaking for 15 minutes. After making up the volume to 100 ml. an aliquot of 1 ml. was taken for analysis. Oestrone was estimated by Brown's method (1952) using the quinol and aqueous sulphuric acid reagent as modified by Bauld (1954).

### *Implantation of Pellets*

Male RIIIf mice about 3 to 4 months old were used because available and proven susceptible to bladder cancer (Armstrong and Bonser, 1944). Jull's (1951) technique for implantation was slightly modified to enable it to be carried out with the least assistance. The head of the mouse was placed nearest the operator. A small piece of sterile linen with a transverse slit in it was laid over the exposed bladder and the bladder then pulled gently through the slit. The linen was then clipped to the cloth on the operating table so as to stretch the slit slightly and thus hold the bladder in position. The linen served as well to sop up urine when the bladder was incised. Before cutting the bladder a holding stitch was put in near the site of the incision about to be made. It was held during the operation by Spencer Wells forceps laid on the table. This became the first stitch for sewing up after insertion of the pellet. A small cut was then made in the bladder. The whole of this operation can be done by one operator. The mice were anaesthetised subcutaneously with a suspension of bromethol in saline.

*Results of long term implants*

1. *Ketosteroid Urine A.*—Implants were made into 20 mouse bladders. 16 mice survived for 25 weeks or longer and 13 of these from 12 to 25 months. Only one bladder tumour was found 18 months after operation. It was a multilobular, sessile, transitional-celled papilloma. Urethral concretions were present but there was no obstruction. The pellet was pigmented, slightly coated and impregnated with salts and its cholesterol content was 0·8 per cent.

2. *Ketosteroid Urine B.*—Implants were made into 17 mouse bladders. 6 mice survived for 20 to 23 weeks. All the remainder died after 25 to 26 weeks from an epidemic of Tyzzer's disease. No bladder tumours were seen.

3. *Oestrone pellets.*—Implants were made into 26 mouse bladders. 16 mice survived for 25 weeks or longer but none for more than 10 months. No bladder tumours were found. All deaths at and after 25 weeks were associated with over-distension of bladders, incontinence and hydronephrosis. The pellets were not moulded into urethral orifices. The bladder distension and hydronephrosis were attributable to atony of bladder musculature induced by oestrogen. Further evidence of absorption of oestrone through the bladder epithelium was obtained by examining the mammae. An average of 5 to 6 rudiments had developed in all these male mice. They were hyperplastic and ducts and alveoli contained a milk-like secretion as is characteristic for this strain in response to natural oestrogens. A few pituitaries were enlarged.

4. *Control wax pellets.*—Implants of wax pellets alone were made into 38 mouse bladders. Thirty-four mice survived to 25 weeks or more and 25 of these from 12 to 25 months. Twelve are still alive after 16 months.

All pellets became coated and impregnated with inorganic salts (calcium, magnesium, ammonium phosphates) but only 3 were appreciably enlarged to form calculi. These were all control wax pellets which had been in the bladders from 1 to 9 months ; 2 were moulded with projections into urethral orifices and had caused obstruction and hydronephrosis. Many pellets became pigmented besides those containing the coloured crude ketosteroid. Some were folded on themselves. No calculi were found in any steroid containing pellets. In a few bladders multiple small calculi had formed. The shape and size of the oestrone pellets were not altered but these were coated as usual with salts. The bladder distension and overflow incontinence associated with them was not accompanied by mechanical obstruction.

Urethral concretions were found in mice with control wax and with ketosteroid pellets. They did not give rise to urinary obstruction. One example of pyonephrosis was seen.

### RESULTS

### *Rate of disappearance of steroids from Pellets*

1. *Ketosteroid*

*Urine of bladder cancer patient* (*A*).—20 pellets weighing about 10 mg. each were inserted. Initially the ketosteroid content of the pellet was 5 per cent, each pellet containing about 0·5 mg. Four of the mice died within 5 days; others lived for 5½ months or more and several for about 2 years. Ketosteroid estimations were carried out on pellets recovered from the bladders of mice post mortem. It was found that the ketosteroid was eliminated completely after 6 to 7 months and in 2 cases negative values were found after 2 and 5 days respectively.

*Urine of prostatic cancer patient* (*B*).—Seventeen pellets were inserted, each weighing approximately 10 mg. and containing 0·4 mg. ketosteroid, which was equivalent to 4 per cent. Sixteen lived more than 5 months but owing to an intercurrent infection all were dead after 6 months. As in the previous case the elimination of the ketosteroid from the mouse bladder was followed. In 7 cases the steroid was completely eliminated after 5½ to 6 months, but in 3 cases after 6 months values of 1 to 1·7 per cent were found.

*Dehydroisoandrosterone.*—In view of the relatively rapid disappearance of the ketosteroid from the wax pellets made from urine extracts when implanted in the mouse bladder, an examination was made of the elimination of dehydroisoandrosterone. This compound, which had been used in standardising the ketosteroid estimation, was made into pellets with wax and tested in the same way as the extracts of urine. The pellets weighed 5 to 10 mg. and contained 20 per cent ketosteroid, i.e. about 1 to 2 mg. The results are recorded in Table I.

2. *Cholesterol*

Initially the cholesterol content of pellets made from steroid extract of Urine B (prostate patient) was 1·9 per cent, i.e. 0·19 mg. per pellet. After 24 months in the mouse bladder the cholesterol remaining in the pellet was 0·4 per cent. In the case of urine from Patient A (bladder) the cholesterol content of the pellet after 14 and 18 months was 0·5 and 0·8 per cent respectively.

Eik-nes, Schellman, Lumry and Samuels (1954) showed that cholesterol was less soluble in buffer solution than other steroids including dehydroisoandrosterone and it was anticipated that its relative insolubility would result in a slower elimination. To confirm this view tests were made with cholesterol pellets. The pellets weighed approximately 10 mg. and each contained about 2 mg. of cholesterol. The findings are shown in Table I.

3. *Oestrone*

The rate of disappearance of oestrone from pellets inserted into mouse bladders has also been examined. As already mentioned, owing to its insolubility this compound could not be made into pellets in the usual manner. Weighed pellets of oestrone however were coated with wax and used in this way. The pellets weighed 5 to 17 mg. and contained 4 to 13 mg. oestrone. As might be expected oestrone was removed relatively slowly from the mouse bladder (Table I).

TABLE I.—*Rate of Disappearance of Steroids from Pellets*

| Duration of experiment in days. | Percentage residual. | | |
|---|---|---|---|
| | Dehydroiso-androsterone. | Cholesterol. | Oestrone. |
| 2 | 78 | — | 91 |
| 3 | 75 | 100 | — |
| 5 | 72 | — | — |
| 6 | — | 97 | 98 |
| 32 | — | — | 97 |
| 33 | — | 97 | — |
| 35 | 20 | — | — |
| 62 | — | 96 | — |
| 63 | 11 | — | 82 |
| 89 | 3 | 78 | — |
| 90 | — | — | 85 |
| 116 | — | — | 77 |

### COMMENT

As a test of ketosteroid action in the bladder the experiment cannot be considered satisfactory on account of the rapid elimination from the pellet. Mice containing oestrone pellets survived too short a time. Evidence of absorption of oestrone from the pellet through the bladder epithelium was unexpected. The possibility that cholesterol might be carcinogenic to bladder epithelium was considered in the light of Hieger's evidence (Hieger, 1949 ; Hieger and Orr, 1954), that it can induce sarcoma in mouse connective tissue. The single papilloma occurred in 1 of 10 mice with crude ketosteroid pellets containing cholesterol which survived for 16 to 25 months, compared with none in 17 with wax alone. An adequate test could only be done with pure cholesterol and larger numbers of mice.

### SUMMARY

Crude ketosteroid fractions from urine of human cancer patients were tested for carcinogenic action in pellets in mouse bladders. The ketosteroids were rapidly eliminated. One papilloma only was found.

Mice implanted with oestrone pellets did not survive longer than 10 months. Oestrone was absorbed from the bladder pellets. No tumours were obtained.

Paraffin pellets alone did not cause tumours.

We are indebted to Mr. Wm. S. Mack and Mr. J. H. McBeath for arranging for the collection of urine from their patients, and to Mrs. R. H. Jack for help with the chemical analyses.

### ADDENDUM.

The 12 remaining control mice died or were killed, between 17 and 20 months. None had bladder tumours.

In a paper published as this article was finished, Boyland and Watson (1956) have recorded the production of bladder papilloma and carcinoma with an endogenous metabolite of tryptophan, 3-hydroxy-anthranilic acid, in bladder pellets. Cholesterol tested in the same way gave negative results.

### REFERENCES

ARMSTRONG, E. C. AND BONSER, G. M.—(1944) *J. Path. Bact.*, **56**, 507.
BAULD, W. S.—(1954) *Biochem. J.*, **56**, 426.
BONSER, G. M., CLAYSON D. B. AND JULL, J. W.—(1951) *Lancet*, ii, 286.
BOYLAND, E. AND WATSON, G.—(1956) *Nature*, **177**, 837.
BROWN, J. B.—(1952) *J. Endocrin*, **8**, 207.
CHALMERS, J. G.—(1951) *Ann. Rep. Brit. Emp. Cancer Campgn*, **29**, 207.
*Idem* AND PULLINGER, B. D.—(1955) *Ibid.*, **33**, 275.
DOBRINER, K., LIEBERMAN, S. AND RHOADS, C. P.—(1948) *J. biol. Chem.*, **172**, 241.
DREKTER, I. J., HEISLER, A., SCISM, G. R., STERN, S., PEARSON, S. AND MCGAVACK, T. H.—(1952) *J. Clin. Endocrin.*, **12**, 55.
DUNNING, W. F. AND CURTIS, M. R.—(1952) *Cancer Res.*, **12**, 702.
*Iidem* AND SEGALOFF, A.—(1947) *Ibid.*, **7**, 511.
EIK-NES, K., SCHELLMAN, J. A., LUMRY, R.AND SAMUELS, L. T.—(1954) *J. biol. Chem.*, **206**, 411.
FOULDS, L.—(1947) *Brit. J. Cancer*, **1**, 172.
HIEGER, I.—(1949) *Ibid.*, **3**, 123.
*Idem* AND ORR, S. F. D.—(1954) *Ibid.*, **8**, 274.
HOLSTI, L. R. AND ERMALA, P.—(1955) *Cancer*, **8**, 679.
HUEPER, W. C., WILEY, F. H. AND WOLFE, H. D.—(1938) *J. industr. Hyg.*, **20**, 46.
JULL, J. W.—(1951) *Brit. J. Cancer*, **5**, 328.
KING, E. J.—(1951*a*) 'Micro-analysis in Medical Biochemistry.', London (Churchill), p. 39. (1951*b*) *Ibid.*, p. 147.
WALPOLE, A. L., WILLIAMS, M. H. C. AND ROBERTS, D. C.—(1955) *Brit. J. Cancer*, **9**, 170.
WILSON, R. H., DEEDS, F. AND COX, A. J.—(1941) *Cancer Res.*, **1**, 595.